# Decreased D2-40 and increased p16^INK4A ^immunoreactivities correlate with higher grade of cervical intraepithelial neoplasia

**DOI:** 10.1186/1746-1596-6-59

**Published:** 2011-07-05

**Authors:** Hongxiu Han, Yan Yang, Zhouping Lu, Qizhi He, Zhenhua Lin

**Affiliations:** 1 Department of Pathology, No. 3 People's Hospital Affiliated to Shanghai Jiaotong University School of Medicine, Shanghai, China; 2 Department of Pathology, Shanghai First Maternity and Infant Hospital, Tongji University School of Medicine, Shanghai, China; 3 Department of Pathology, Yanbian University School of Medicine, Yanji, China

**Keywords:** D2-40, cervical intraepithelial neoplasia, immunohistochemistry, p16^INK4A^

## Abstract

**Background:**

D2-40 has been shown a selective marker for lymphatic endothelium, but also shown in the benign cervical basal cells. However, the application of D2-40 immunoreactivity in the cervical basal cells for identifying the grade of cervical intraepithelial neoplasia (CIN) has not been evaluated.

**Methods:**

In this study, the immunoreactive patterns of D2-40, compared with p16^INK4A^, which is currently considered as the useful marker for cervical cancers and their precancerous diseases, were examined in total 125 cervical specimens including 32 of CIN1, 37 of CIN2, 35 of CIN3, and 21 of normal cervical tissue. D2-40 and p16^INK4A ^immunoreactivities were scored semiquantitatively according to the intensity and/or extent of the staining.

**Results:**

Diffuse D2-40 expression with moderate-to-strong intensity was seen in all the normal cervical epithelia (21/21, 100%) and similar pattern of D2-40 immunoreactivity with weak-to-strong intensity was observed in CIN1 (31/32, 97.2%). However, negative and/or focal D2-40 expression was found in CIN2 (negative: 20/37, 54.1%; focal: 16/37, 43.2%) and CIN3 (negative: 22/35, 62.8%; focal: 12/35, 34.3%). On the other hand, diffuse immunostaining for p16^INK4A ^was shown in 37.5% of CIN1, 64.9% of CIN2, and 80.0% of CIN3. However, the immunoreactive pattern of D2-40 was not associated with the p16^INK4A ^immunoreactivity.

**Conclusions:**

Immunohistochemical analysis of D2-40 combined with p16^INK4A ^may have a significant implication in clinical practice for better identifying the grade of cervical intraepithelial neoplasia, especially for distinguishing CIN1 from CIN2/3.

## Background

Although the histological assessment of cervical biopsies is often considered as the "gold standard", evaluating the grade of cervical intraepithelial neoplasia (CIN) by conventional light microscopy, especially distinguishing CNI1 from CIN2/3, often presents a diagnostic issue in surgical pathology [1]. There has been much recent attention regarding use of p16 immunoreactivity for the detection of high grade cervical squamous lesions, however, assessment of its clinical applications is seriously hampered by lack of standardized methodology [2]. Novel markers are needed to apply on histological specimens to identify the grade of cervical intraepithelial neoplasia when the lesion is morphologically difficult to assess, especially between CIN1 and CIN2/3.

D2-40 is a recently developed, commercially available monoclonal antibody directed against M2A antigen, a *M*_r _40 000 surface sialoglycoprotein originally detected in association with germ cell neoplasia and fetal testicular gonocytes [3]. Since D2-40 has also been demonstrated selective immunoreactivity for lymphatic endothelium [4], its proposed clinical uses include demonstration of lymphatic invasion by primary tumors and its use as a marker of certain vascular lesions [5,6]. Besides the above, the D2-40 immunostaining has been observed in malignant mesothelioma [7], carcinoma of the uterine cervix and benign cervical squamous epithelia [8].

p16^INK4A ^is currently used as a 'positive' immunohistochemical marker for CIN, which is proposed to aid the identification of high-grade cervical lesions [9]. To evaluate the use of D2-40 in helping the diagnosis of CIN, we performed immunoreactivity of D2-40, compared to p16^INK4A^, on cervical specimens to aid a better identification of grade of CIN.

## Materials and methods

### Clinical specimens

Cases were retrieved from the files of the Departments of Pathology in Shanghai Jiaotong University and Tongji University. This study consisted of 125 cases of CIN1 (n = 32), CIN2 (n = 37), CIN3 (n = 35) and normal cervical tissue (n = 21). The consensus diagnosis was confirmed by an expert pathology panel when inter-observer variability in grading CIN based solely on H&E-stained slides occurred. One representative paraffin block from each case was used for the study.

### Immunohistochemistry

Immunohistochemical assays were performed on formalin-fixed paraffin-embedded tissues. Sections (5 μm thick) were cut and deparaffinized in xylene and rehydrated in graded alcohols. Slides were boiled in citrate buffer (pH 6.0) at 95 ~ 100°C for 5 min and were cooled down for 20 min. Endogenous peroxide was blocked by 3% hydrogen peroxide in methanol for 10 min. Sections were incubated with D2-40 monoclonal antibody (1:200, DAKO, Carpinteria, CA, USA) and monoclonal anti-p16^INK4A ^antibody (clone G175-405, DAKO, Carpinteria, CA, USA) for 1 h at 37°C. Immunohistochemical staining was performed using EnVision + HRP DAB system (DAKOCytomation, Carpinteria, CA, USA). All sections were counterstained with Meyer's Hematoxylin. The sections processed without the primary antibodies were used as negative control.

### Immunohistochemical evaluation

Immunohistochemical D2-40 reactivity was evaluated as the cytoplasmic staining in basal cells of squamous epithelium. D2-40 expression was scored semiquantitatively as previously described [8] as follows: (-), 0% of immunoreative cells; (+), <5% of immunoreactive cells with weak staining; (++), 5 ~ 50% of immunoreactive cells with weak to moderate staining; (+++), >50% of immunoreactive cells with moderate to strong staining. The pattern of D2-40 expression was evaluated as follows: negative (-); focal expression (+ ~ ++): diffuse expression (+++). p16^INK4A ^expression was evaluated according to the criteria previously established by Klaes et al [10] as follows: negative (<1% of the cells were positive); focal expression (isolated cells or small cell clusters, but <25% of the cells were positive); diffuse expression (>25% of the cells were positive).

Two investigators evaluated the specimens independently on separated counts. The results from the two investigators were highly correlated (r > 0.85 for all counts). Calculations were therefore done using averages of the two sets of counts.

### Statistical analysis

Data were presented as absolute numbers and percentages. χ^2^-test for nominal data was used to compare baseline characteristics. Reported *P*-values < 0.05 were considered as significant.

## Results

**Figure 1:**
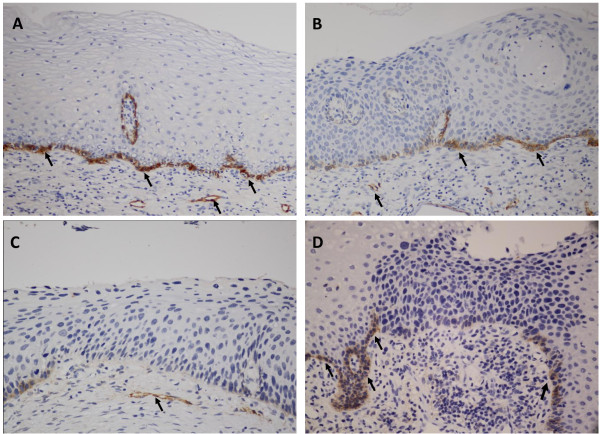
**The expression of D2-40 in the normal cervical tissue and CIN**. The D2-40 immunoreactivity was localized to the basal cell layer as well as the endothelial cells of lymphatic vessels indicated by arrow. A: diffuse expression of D2-40 in the basal cells of squamous epithelium in the normal cervical tissue. B: diffuse expression of D2-40 in the basal cells of squamous epithelium in CIN1 on the right and foal expression of D2-40 in the basal cells of squamous epithelium in CIN2 on the left. C: negative expression of D2-40 in CIN2. D: negative expression of D2-40 in CIN3 in the middle and diffuse expression of D2-40 in the basal cells of squamous epithelium in the normal cervical tissue indicated by arrow.

D2-40 protein was expressed in cytoplasm of the basal cells of squamous epithelium (SE) as well as in the epithelial cells of lymphatic vessels in the cervical stroma (Figure 1). Diffuse expression of D2-40 with moderate-to-strong staining intensity was seen in the basal cells of all the normal cervical tissues (21/21, 100%), and similar pattern of D2-40 expression was shown in CIN1 (diffuse: 31/32, 97.2%). However, negative and/or focal expression of D2-40 was found in CIN2 (negative: 20/37, 54.1%; focal: 16/37, 43.2%) and CIN3 (negative: 22/35, 62.8%; focal: 12/35, 34.3%). Significant difference in D2-40 expression was observed between CIN1 and CIN2/3 (p < 0.01), while no significant difference in D2-40 expression was observed between normal cervix and CIN1, and between CIN2 and CIN3 (Table 1).

**Table 1:** The expression of D2-40 in the cervical intraepithelial neoplasia (CIN)

		D2-40 expression (%)			
**Lesion type**	**n**	**negative**	**focal**	**diffuse**	**Positivity**

**Normal cervix**	21	0 (0)	0 (0)	21 (100%)	100%
**CIN 1**	32	0 (0)	1(2.8%)	31 (97.2%)	100%
**CIN 2**	37	20 (54.1%)	16 (43.2%)	1 (2.7%)	45.9%
**CIN 3**	35	22 (62.8%)	12 (34.3%)	1 (2.9%)	37.1%

**Figure 2:**
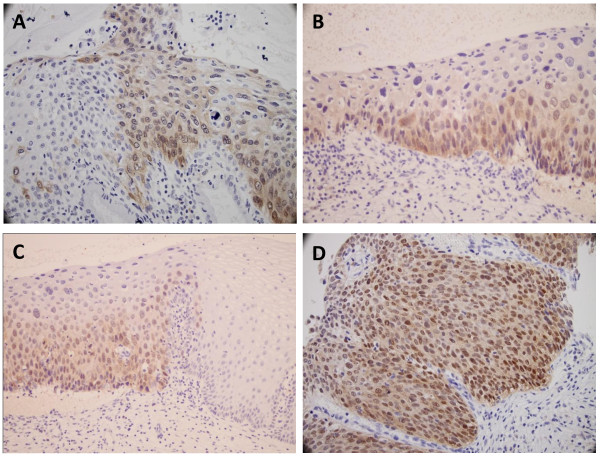
**Representative examples of p16**^**INK4A **^**immunoreactive patterns in the normal cervical tissue and CIN**. A: focal p16^INK4A ^immunostaining in CIN1. B: diffuse p16^INK4A ^immunostaining restricted to the lower third of the cervical epithelium in CIN1. C: diffuse p16^INK4A ^immunostaining of more than a third of the cervical epithelium in CIN2 on the left and negative p16^INK4A ^immunostaining in the normal cervical tissue on the right. D: diffuse p16^INK4A ^immunostaining of full thickness of the cervical epithelium in CIN3.

Twenty-one cases of normal cervical tissue did not show any immunoreactivity for p16^INK4A^, meanwhile, dysplastic epithelium showed cytoplasmic and/or nuclear staining for p16^INK4A ^in 98.1% (102/104) of CIN. There was a grade-dependent fashion of diffuse p16^INK4A ^immunostaining shown in 37.5% of CIN1, 64.9% of CIN2 and 80% of CIN3 (Table 2). Diffuse p16^INK4A ^immunostaining was often restricted to the lower third of the cervical epithelium in CIN1 and more than a third/full thickness of the cervical epithelium in CIN2/3 (Figure 2).

**Table 2:** The expression of p16^INK4A ^in the cervical intraepithelial neoplasia (CIN)

		**p16**^**INK4A **^**expression (%)**			
**Lesion type**	**n**	**negative**	**focal**	**diffuse**	**Positivity**

**Normal cervix**	21	21 (100%)	0 (0)	0 (0)	0
**CIN 1**	32	2 (6.2%)	18 (56.3%)	12 (37.5%)	93.8%
**CIN 2**	37	0 (0)	13 (35.1%)	24 (64.9%)	100%
**CIN 3**	35	0 (0)	7 (20.0%)	28 (80.0%)	100%

Diffuse and focal/negative immunostaining of D2-40 correspond to CIN1 (31/32) and CIN2/3 (70/72), accordingly. In contrast, increased diffuse immunostaining compared to decreased focal immunostaining of p16^INK4A ^was associated with higher grade of CIN. However, not only the present of D2-40 in CIN1 was not associated with p16 ^INK4A ^negativity or non-block positivity, but also the loss of D2-40 in CIN2/3 was not associated with block positivity for p16 ^INK4A^.

## Discussion

Histological evaluation remains a basis for treatment and follow-up of women with CIN. The fundamental premise for treating or following young women with CIN hinges on the risk of CIN2 or CIN3 for which cone biopsy or LEEP will be required. Hence, helpful biological markers are in need of distinguishing CIN1 from CIN2/3 when the diagnosis is not certain, particularly in young women.

The monoclonal antibody D2-40 was described to react with a novel oncofetal membrane antigen M2A, presenting on fetal gonocytes, intratubular germ-cell neoplasia and seminoma cells [11]. The M2A antigen has also been shown as a developmental marker for human Sertoli cells, presenting on immature Sertoli cells until puberty, and loosing during their transition to a mature adult phenotype [12]. We found that D2-40 immunoreactivity was observed exclusively in the basal cell layer of the cervical squamous epithelium, which agrees with the previous study [8]. Taken together, D2-40 protein expression may be predominantly associated with immaturity. D2-40 has been served as a new selective marker for lymphatic endothelium, and used in identifying the presence of lymphatic invasion in various malignant neoplasms [4], including cervical carcinoma as well as cervical neoplasia [13]. The D2-40 immunoreactivity observed in the lymphatic endothelium of the cervical stroma in this study acts as an internal control, indicating that the loss of D2-40 protein expression in the squamous epithelium in CIN2/3 specimens is not a false negative pattern arising from fixation or processing issues. Furthermore, this study expanded the information that the decreased expression of D2-40 in the basal cells of SE correlates the grade of CIN, especially showing the significant difference between CIN1 and CIN2/3. Likewise, low D2-40 immunoreactivity correlates with lymphatic invasion and nodal metastasis in early-stage squamous cell carcinoma of the uterine cervix [8], and D2-40 positivity in tumor cells is associated with a better prognosis in ASC [13]. Thus, the D2-40 protein may be a better prognostic marker in cervical lesion. That might be associated with the M2A, recognized by the D2-40 antibody. Although its function is yet unclear, it contains one of the mucin-type glycoproteins that are expressed on human normal cells and tumors [14,15]. Mucins are large, highly glycosylated proteins recognized by their tandem repeat domains, first as components of cell surfaces, and later for their roles in the protection of epithelia and other cells [16,17].

In the current study, we also compared the D2-40 immunoreactivity to p16^INK4A^, identified as a biomarker for transforming human papillomavirus (HPV) infections. Increased cases with diffuse immunostaining of p16^INK4A ^occurred in the higher grade of CIN (CIN2/3), which is in agreement with the previous studies [18-20]. In addition, several studies suggest an improved diagnostic accuracy for diagnosing CIN lesions with the diffuse p16^INK4A ^immunoreactivity [21,22]. Although there is good evidence that diffuse p16^INK4A ^immunostaining correlates with the severity of CIN, we have to take into consideration the limited specificity of p16^INK4A ^immunoreactivity. Because the focal staining was seen in more than a half of CIN1 cases, but this pattern was also seen in some CIN2/3 cases. Thus, an additional ideal biomarker needs to be explored. This study shows that the immunoreactive pattern of D2-40 was more specific than that of p16^INK4A ^immunoreactivity when distinguishing CIN1 from CIN2/3 because diffuse and focal/negative immunostaining of D2-40 predominantly presents in CIN1 and CIN2/3, respectively. No correlation between D2-40 and p16^INK4A ^immunoreactity was found, which might be due to p16^INK4A ^associated with transforming HPV infections, but not D2-40. Although no correlation between D2-40 and p16^INK4A ^immunoreactity was shown, the data in the current study indicate that the combined use of D2-40 and p16^INK4A ^immunoreactivities in routine histopathology would improve accuracy of diagnosis of CIN.

## Conclusions

D2-40 may be a helpful marker for distinguishing CIN1 from CIN2/3 in pathological practice.

## Competing interests

The authors declare that they have no competing interests.

## Authors' contributions

HH and ZL^3 ^designed the study and wrote the manuscript. YY and ZL^2 ^performed immunohistochemistry. HH and QH performed count and statistical analysis. In cases of discrepant assessments, HH, YY and QH discussed to obtain an agreement. All authors read and approved the final manuscript.
